# Knockdown of CD44 inhibits proliferation, migration and invasion of osteosarcoma cells accompanied by downregulation of cathepsin S

**DOI:** 10.1186/s13018-022-03048-x

**Published:** 2022-03-09

**Authors:** Lingwei Kong, Hairu Ji, Xintian Gan, Sheng Cao, Zhehong Li, Yu Jin

**Affiliations:** 1 Department of Orthopaedics, The Affiliated Hospital of Chengde Medical College, No. 1 Nanyingzi Street, Chengde, 067000 Hebei China; 2 Pathology Teaching and Research Section, Chengde Medical College, Chengde, 067000 Hebei China

**Keywords:** Osteosarcoma, CD44, Cathepsin S, Proliferation, Migration, Invasion

## Abstract

**Background:**

Osteosarcoma (OS) is a malignant bone tumour of mesenchymal origin. These tumours are characterised by rich vascularisation, therefore promoting rapid proliferation and facilitating metastasis. CD44 has been reported to be involved in OS, but its role and molecular mechanisms in the pathogenesis of the disease are not fully determined.

**Methods:**

In this study, we investigated the antitumor effect of CD44 on the development of OS and further explored the molecular mechanisms. The expression of CD44, cathepsin S and MMP-9 was detected by Western blot (WB) and reverse transcription-polymerase chain reaction (RT-qPCR) in different cell lines (MG63, U2OS OS and hFOB 1.19). To elucidate the role of CD44 in OS, MG63 and U2OS cells were treated with small interference RNA (siRNA) to knock down CD44, and the knockdown efficiency was validated with GFP and RT-qPCR. Furthermore, cell proliferation was assayed using Cell Counting Kit‑8 (CCK-8) and colony formation assays, and cell migration and invasion were assayed by transwell and wound-healing assays.

**Results:**

We found that CD44 expression in the MG63 and U2OS OS cell lines was markedly increased compared to that of the human osteoblast hFOB 1.19 cell line. Knockdown of CD44 inhibited proliferation, migration and invasion of MG63 and U2OS cells. Cathepsin S expression in the MG63 and U2OS OS cell lines was increased compared to that of the human osteoblast hFOB 1.19 cell line. When CD44 was knocked down, its expression level went down.

**Conclusion:**

Taken together, our data reinforced the evidence that CD44 knockdown inhibited cell proliferation, migration and invasion of OS cells accompanied by altered expression of cathepsin S. These findings offer new clues for OS development and progression, suggesting CD44 as a potential therapeutic target for OS.

## Introduction

Osteosarcoma (OS) is the most common primary bone tumour, mainly occurring in children and adolescents, and the third most frequent in adults, following chondrosarcoma and chordoma. The overall incidence of OS is 3.4 per million cases per year worldwide [1], and the principal cause of death in patients suffering from OS is pulmonary metastasis [2, 3]. Osteosarcoma is a primary bone cancer characterised by cancer cells that produce calcified osteoid extracellular matrix and inducing lung metastases with a high frequency [4]. Despite recent advances in treating osteosarcoma with a combination of chemotherapy and surgery, the 5-year survival rate remains low, and the prognosis for patients is poor [5]. The cellular and molecular mechanisms underlying the progression of osteosarcoma, including the rate of cancer cell proliferation, the formation of metastatic lesions and the development of drug resistance, remain unclear.

Cluster of differentiation 44 (CD44) is a complex transmembrane adhesion glycoprotein considered an essential bridge molecule as it links the extracellular matrix and intracellular skeletal proteins and participates in intracellular signal transduction, affecting cell deformation or movement through cytoskeletal changes [6]. Numerous studies have reported that CD44 not only participates in normal cellular functions but also plays pivotal roles in pathological processes [7]. For example, CD44-RhoA-YAP signalling mediates mechanics-induced fibroblast activation, and targeting this pathway could ameliorate crystalline silica-induced silicosis and provide a potential therapeutic strategy to mitigate fibrosis [8]. It is noteworthy that CD44 expression was found upregulated in different tumours [9–11], promoting cancer cell invasion and migration [12, 13]. However, the role and molecular mechanisms of CD44 in the development and progression of OS remain uncertain.

Cathepsin S (CTSS), a lysosomal cysteine protease of papain subfamily, has a series of functions under extracellular conditions, unlike other family members [14]. Like matrix metalloproteinases, members of the histone family have been associated with metastasis and cancer recurrence [15]. Cathepsin S is highly expressed in renal clear cell carcinoma [16], hepatocellular carcinoma [17], cervical cancer [18], lung cancer [19] and other tumours and is an essential regulator of tumour growth and invasion. Suppression of cell migration and invasion by modulation of Ca^2+^-dependent downstream effectors after CTSS inhibition [20]. The expression of cathepsin S was regulated by PI3K/Akt and Ras/Raf/MAPK signalling pathways, is a candidate target for blocking the metastasis of breast and oral cancers [21, 22]. It is evident that CTSS is highly correlated with tumour invasion and migration and plays a pro-cancer role in most tumours.

The present study analysed the CD44 expression pattern in OS cell lines using reverse transcription‑quantitative PCR (RT‑qPCR) and Western blot (WB). Furthermore, loss‑of‑function experiments were performed to investigate the biological roles of CD44 in OS. The results revealed that CD44 expression was upregulated in OS cell lines. In addition, in vitro assays revealed that CD44 downregulation inhibited cell proliferation, migration, and invasion, probably by regulating cathepsin S. These findings suggest that CD44 functions as an oncogene and future research may contribute to the development of new tools for the diagnosis and treatment of OS.

## Materials and methods

### Cell culture

The human OS MG63 and U2OS cell lines were purchased from the Cell Bank of Shanghai Institute of Cell Biology (Shanghai, China) and cultured in modified Eagle’s medium (MEM, Gibco) supplemented with 10% fetal bovine serum (FBS, Gibco) at 37 °C with 5% CO_2_.

The normal human osteoblastic cell line hFOB 1.19 was purchased from the Cell Bank of Shanghai Institute of Cell Biology (Shanghai, China) and maintained in D-MEM/F-12 (Gibco) supplemented with 10% FBS (Gibco) and 0.3 mg/mL Geneticin (G418; Gibco) at 37 °C with 5% CO_2_.

### Small interference RNA transfection

Small interference RNA (siRNA) for transfection was purchased from Ribobio (Guangzhou, China). Transfections (50 nM final concentration of siRNA) were performed using Invitrogen Lipofectamine 2000 (Thermo Fisher Scientific) following the protocols of the manufacturer. Three different siRNAs (si-CD44-1, si-CD44-2, si-CD44-3) were tested, and si-CD44-1 and si-CD44-2 were selected for subsequent experiments. A control siRNA (si-NC) was used in all the experiments.

### RT‑qPCR analysis

MG63 and U2OS cells were treated with si-CD44 or si-NC for 24 h, and total RNA was extracted from the OS cell lines and the normal human osteoblastic cell line hFOB 1.19 using Trizol (Supersmart, China). Next, 2 μg of RNA was used to synthesise the complementary DNA (cDNA) by reverse transcriptase (ABclonal, China). The resulting complementary cDNA was used for PCR analysis. The relative levels of genes were detected by RT-qPCR using SYBR Premix Ex Taq™ (ABclonal, China). The PCR cycling conditions were 95 °C for 5 min, followed by denaturation for 10 s at 95 °C and extension for 20 s at 60 °C for 40 cycles. GAPDH was used as an internal loading control. All reactions were performed in triplicates. Fold changes were calculated using the 2^−ΔΔCq^ method. The primers were as follows: CD44 forward, 5′-GAGCAGCACTTCAGGAGGTT-3′ and reverse, 5′-TGGTTGCTGTCTCAGTTGCT-3′; cathepsin S forward, 5′-GCAGTGGCACAGTTGCATAA-3′ and reverse, 5′-AGCACCACAAGAACCCATGT-3′; GAPDH forward, 5′-GTCTCCTCTGACTTCAACAGCG-3′ and reverse, 5′-ACCACCCTGTTGCTGTAGCCAA-3′.

### Western blot analysis

MG63 and U2OS cells were treated with si-CD44 or si-NC for 48 h, and total proteins were extracted using RIPA buffer containing protease inhibitor cocktail. Protein concentrations were determined using the BCA Protein Assay (Multi sciences). Proteins (30 µg/lane) were separated by 10% SDS-PAGE and transferred to PVDF membranes. The membranes were blocked with 5% non-fat milk for 2 h at room temperature (RT). Next, the membranes were incubated with anti-CD44 (1:2000, ABclonal, China), cathepsin S (1:2000, Affinity, China), anti-MMP-9 (1:2000, ABclonal, China) at 4 °C overnight. Subsequently, the appropriate horseradish peroxidase (HRP)-linked secondary antibodies (1:5000, Sera care) were used to visualise the immunoreactivity. GAPDH was used as an internal control. The intensity of each band was measured with ImageJ.

### Cell counting Kit‑8 (CCK‑8) assay

MG63 and U2OS cells were treated with si-CD44 or si-NC for 24 h. Cells were prepared into suspension and MG-63, and U2OS cells were seeded in 96-well plates at a density of 1 × 10^3^ cells per well and incubated in a humidified incubator at 37 °C for 24, 48, 72 h and 96 h. Subsequently, the cells were incubated with 10 µl CCK-8 solution for another 1 h at 37 °C. Optical density (OD) was determined at a wavelength of 450 nm.

### Colony formation assay

MG63 and U2OS cells were treated with CD44 siRNA or negative control for 24 h. Cells were then resuspended and seeded in 6-well plates at a density of 500 cells per well and cultured for 15 days. Subsequently, cells were fixed with pre-cooled methanol for 30 min at RT and stained with 0.1% crystal violet for 20 min at RT, washed twice with PBS and twice with double distilled water. The colonies were counted and analysed under a light microscope.

### Wound-healing assay

To evaluate the role of CD44 in OS cell migration, MG63 and U2OS cells were transfected with si-CD44 or si-NC for 24 h. Cells were resuspended and seeded in 6-well plates at a density of 1 × 10^6^ cells per well, and 2 ml of culture medium supplemented with 10% FBS was added. Cells were grown to 90% confluence, and then, a uniform and consistent wound was scraped on the bottom of the 6-well plate with a 200 μL plastic pipette tip (time set as 0 h). PBS was used to remove floating cells. Subsequently, cells were incubated in fresh complete medium (1% FBS) for 0, 24 and 48 h and the number of migrated cells were observed and counted under a light microscope.

### Transwell assay

Migration and invasion abilities of MG-63 and U2OS cells were measured using a transwell assay. The Matrigel was incubated at 37˚C for 5 h before testing. OS cells were transfected with si-CD44, si-cathepsin S or si-NC for 24 h. 1 × 10^5^ transfected cells were resuspended in serum-free medium and seeded in the upper chamber with or without Matrigel (BD Biosciences) for the invasion and migration assays, respectively. Subsequently, medium containing 20% FBS was added to the lower chambers. Following a 24 h incubation, the cells from the upper compartments were scraped off with cotton swabs, while the cells that migrated to or invaded the lower surface of the membrane were fixed with pre-cooled methanol at RT for 20 min and stained with 0.1% crystal violet at RT for 20 min. The stained cells were counted in five random fields off view under a light microscope at × 200 magnification, and all experiments were repeated three times.

### Statistical analysis

The results are presented as the mean ± SD. Statistical analyses were performed using SPSS 23.0 (IBM Corp, USA) and GraphPad Prism 9.0 (La Jolla, CA, USA) software. ANOVA test was applied to compare differences among multiple groups. *P* < 0.05 was considered to indicate a statistically significant difference.

## Results

**Fig. 1:**
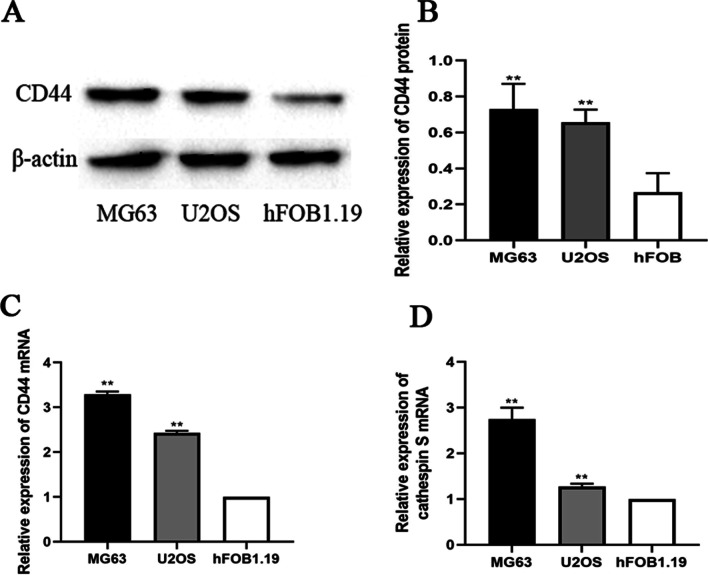
CD44 and cathepsin S are upregulated in OS cell lines. **A**, **B** CD44 protein levels in MG63, U2OS, and hFOB 1.19 cell lines. **P < 0.01 versus hFOB group. **C** CD44 mRNA levels in MG63, U2OS, and hFOB 1.19 cell lines. **P < 0.01 versus hFOB group. **D** Cathepsin S mRNA levels in MG63, U2OS, and hFOB 1.19 cell lines. **P < 0.01 versus hFOB group

**Fig. 2:**
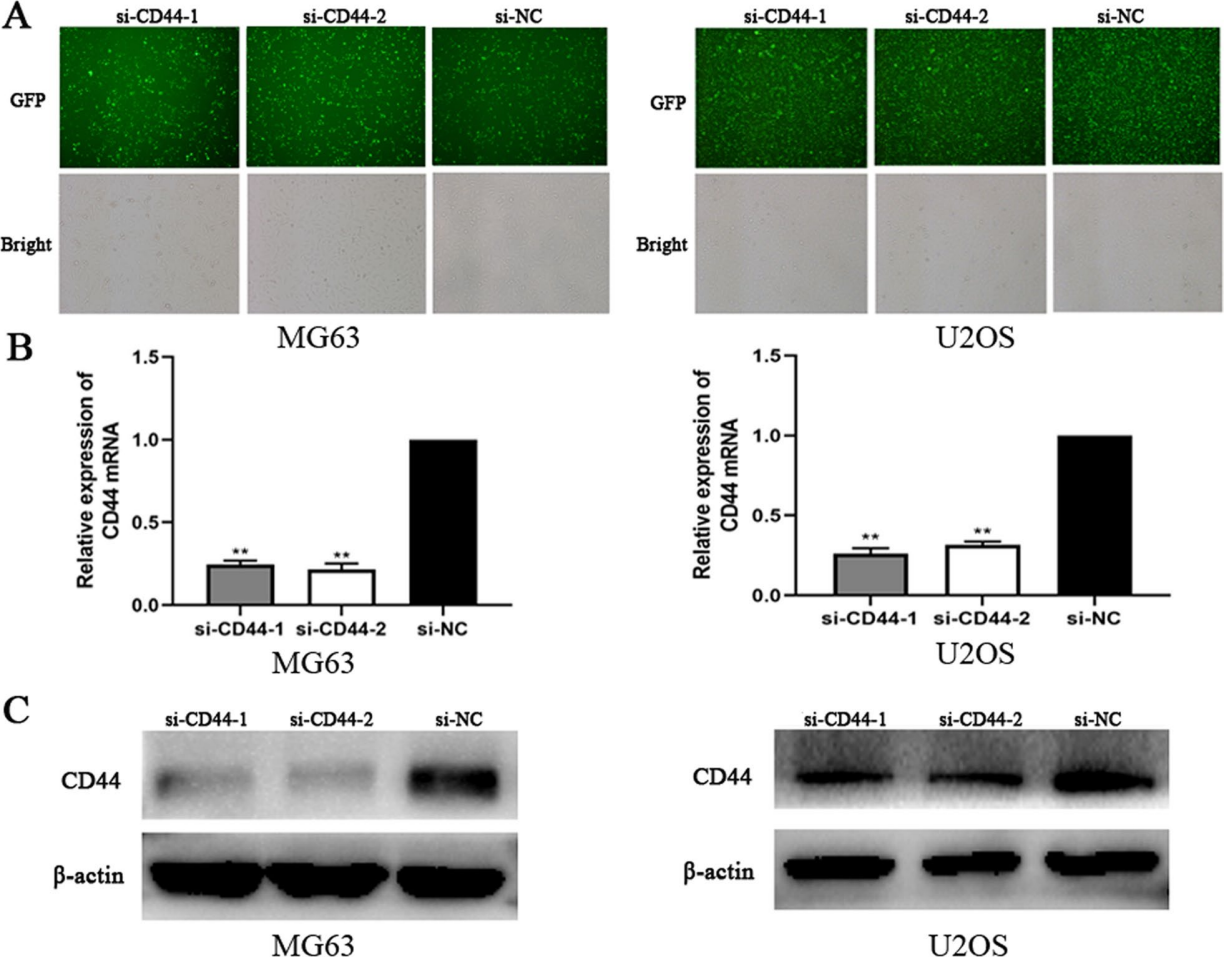
CD44 knockdown in MG63 and U2OS cells in vitro. **A** Transfection efficiency of MG‑63 and U2OS cells was assessed by fluorescence microscopy. GFP, green fluorescent protein. Magnification, × 200. **B** Reverse transcription‑quantitative PCR analysis was used to assess the mRNA expression levels of CD44 in MG‑63 and U2OS cells after transfection for 24 h. **P < 0.01 versus si-NC group. **C** Western blot was used to assess CD44 expression levels in MG‑63 and U2OS cells 48 h after transfection

**Fig. 3:**
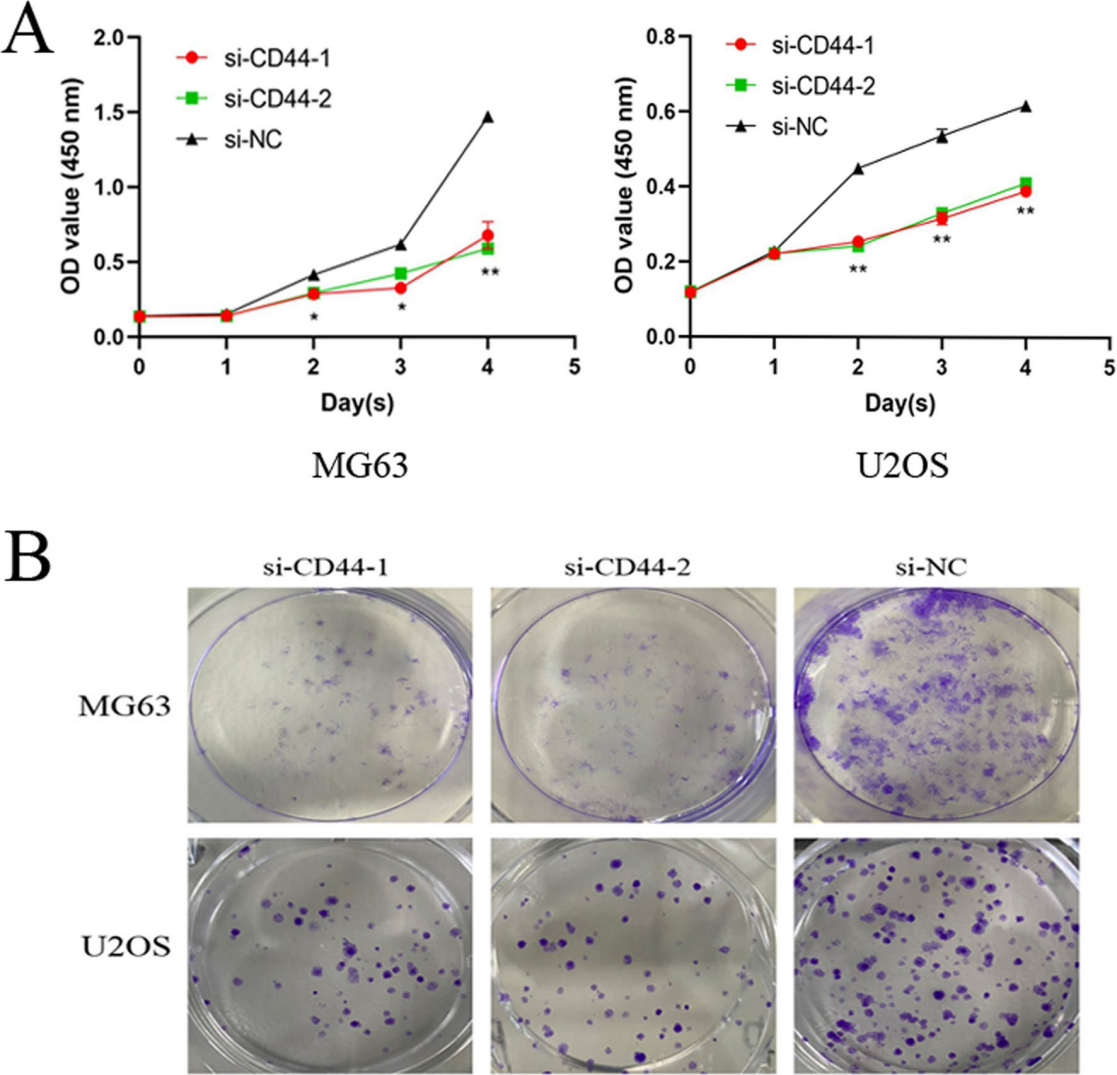
CD44 knockdown inhibited the proliferation of MG63 and U2OS cells. **A** CCK8 assay demonstrated that silencing of CD44 inhibited the cell proliferation capability on the indicated time points after transfection with CD44 siRNA (si-CD44). *P < 0.05, **P < 0.01 versus si-NC group (the significant differences between the si-CD44-1, si-CD44-2 group and the si-NC group were consistent, with a common label). **B** Colony formation in MG-63 and U2OS cells. All data are presented as the mean ± SD of *n* = 3 experiments

**Fig. 4:**
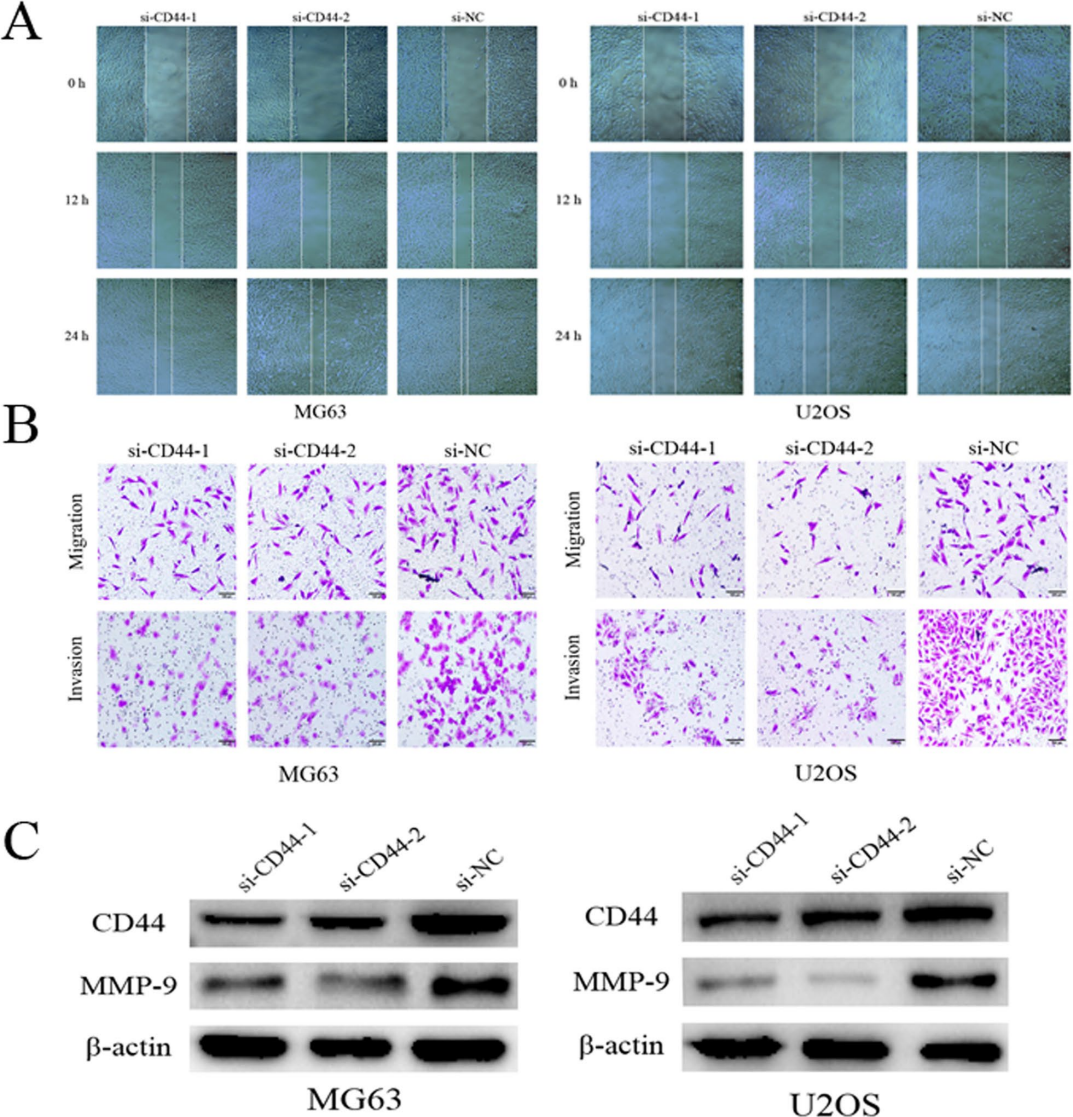
CD44 knockdown inhibited the migration and invasion of MG63 and U2OS cells. **A** A wound-healing assay was performed to detect the migration of MG-63 and U2OS cells. **B** Transwell assay was performed to detect migration and invasion of MG-63 and U2OS cells. Magnification, × 200. **C** Expression levels of migration and invasion-related proteins (MMP-9) were detected by Western blotting

**Fig. 5:**
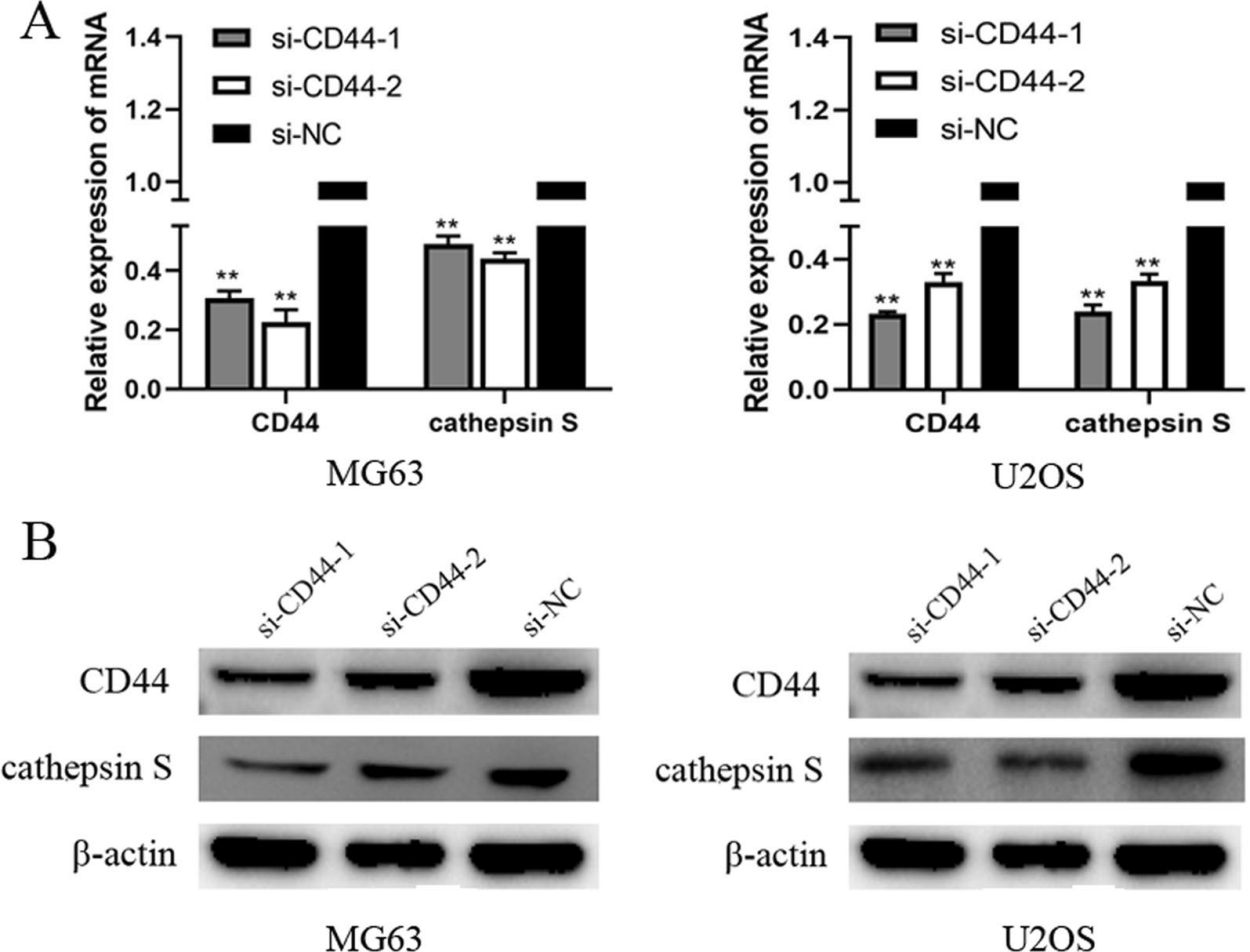
CD44 knockdown downregulated the expression of cathepsin S in MG63 and U2OS cells. **A** mRNA expression levels of CD44, cathepsin S, and β-actin were detected by RT-qPCR. **B** Protein expression levels of CD44, cathepsin S, and β-actin were detected by Western blotting. **P < 0.01 versus control group

1. CD44 is upregulated in OS cell lines. The present study first examined CD44 expression levels in the MG63, U2OS, and hFOB 1.19 cell lines by WB and RT-qPCR. Compared with the hFOB 1.19 cell line, the expression of CD44 was markedly upregulated in the OS cell lines (Fig. 1A–C).
2. CD44 knockdown in MG63 and U2OS cells in vitro. MG63 and U2OS cells were transfected with si-CD44 or si-NC for 24 h, and the transfection efficiency was detected using fluorescence microscopy (Fig. 2A). CD44 mRNA and protein expression was quantified by RT-PCR and Western blot, respectively, after CD44 knockdown in MG63 and U2OS cells. As shown in Fig. 2B,C, the results revealed that the siRNA transfection decreased CD44 expression, but as expected, no significant difference was observed between the si-NC and control groups. For subsequent experiments, two (siCD44-1, siCD44-2) of the three CD44 siRNA with high transfection efficiency were selected.
3. CD44 knockdown inhibited the proliferation of MG63 and U2OS cells. To assess the role of CD44 in MG63 and U2OS cell proliferation, siRNA was transfected to silence CD44 expression. Subsequently, cell proliferation was assessed using CCK-8 and colony formation assays. As demonstrated by the result of the CCK-8 assay, cell growth was suppressed in CD44-silenced MG63 and U2OS cells compared with the si-NC-transfected cells (Fig. 3A). In addition, the colony formation ability of si-CD44-transfected cells was decreased (Fig. 3B). These results revealed that downregulation of CD44 markedly decreased the proliferation of MG63 and U2OS cells.
4. CD44 knockdown inhibited the migration and invasion of MG63 and U2OS cells. To investigate the role of CD44 in the migration and invasion of OS cells, the wound-healing assay was used at 0, 12, or 24 h after transfection. The result of the wound-healing assay showed that the migration distances of cells transfected with si-NC were compared to the migration distances in CD44-silenced cells (Fig. 4A).
  The result of the transwell migration and invasion assay showed that the number of control si-NC-transfected cells was more than the number of CD44-silenced cells (Fig. 4B). Furthermore, Western blot was applied to evaluate the matrix metalloproteinase MMP-9 protein levels. As shown in Fig. 4C, silencing of CD44 decreased MMP-9 expression in MG-63 and U2OS cells compared with the si-NC group. Therefore, the results suggested that the migration and invasion abilities of MG-63 and U2OS cells were suppressed following CD44 knockdown.
5. Cathepsin S is upregulated in OS cell lines. The expression level of cathepsin S in the MG63, U2OS, and hFOB 1.19 cell lines by RT-qPCR. Compared with the hFOB 1.19 cell line, the expression of cathepsin S was upregulated in the OS cell lines (Fig. 1D).
6. CD44 knockdown downregulated the expression of cathepsin S in MG63 and U2OS cells. To further confirm the underlying mechanism of CD44 in OS, we detected the mRNA and protein expression of cathepsin S by RT-PCR and Western blot after CD44 knockdown in MG63 and U2OS cells. The mRNA and protein levels of cathepsin S in the CD44-silenced OS cells were markedly reduced compared with the control cells (si-NC) at 24 and 48 h after transfection (*p* < 0.01) (Fig. 5A,B). These data indicated that CD44 exerted its effects in OS in part by regulating cathepsin S.

## Discussion

At present, the molecular mechanisms underlying the development of OS have not been fully explored. Therefore, it is crucial to elucidate the predictive markers of OS and their potential regulatory mechanisms. CD44, also known as homing cell adhesion molecule, is a cell surface transmembrane glycoprotein molecule involved in cell–cell and cell–extracellular matrix communication. In humans, CD44 proteins are encoded by a highly conserved gene located on the short arm of chromosome 11 (11p13) [23], whose expression is elevated in a wide range of malignant tumours [24], such as colon tumours, ovarian clear cell carcinoma and glioblastoma. In this study, we found that CD44 was highly expressed in osteosarcoma cell lines compared to hFOB1.19 human osteoblasts.

Previous studies on the role of CD44 in osteosarcoma mostly used it as a cancer stem cell marker to evaluate the stem cell status of osteosarcoma [25, 26], or it could be used as an evaluation protein for pulmonary metastasis of osteosarcoma [27, 28]. In our experiment, the effect of CD44 knockdown on biological behaviour of osteosarcoma was directly observed. CCK8 and Colony formation assays showed that down-regulated CD44 inhibited the proliferation of OS cells, wound-healing and transwell assays showed that down-regulated CD44 inhibited the invasion and migration of OS cells. MMP-9 can cleave many extracellular matrix (ECM) proteins and regulate ECM remodelling, which can be used as a marker of tumour invasion and metastasis, and its expression level is positively correlated with tumour invasion and metastasis [29]. Many studies have used MMP-9 as a regulatory factor for evaluating tumour invasion and metastasis (including osteosarcoma) [30–32]. Both matrix metalloproteinases and CD44 are critical for tumour invasion. MMP-9 has been shown to be associated with CD44 on breast cancer cells [33] and human melanoma cells [34]. Mt1-MMP can cleave CD44 and promote cell migration in pancreatic tumour cell lines [35]. Previous studies have shown that, the activated Ras-MEK-ERK signalling pathway can modulate the transcriptional expression of MMP-9 [36], which can interact with the CD44 extracellular domain, this interaction allows the secretion and activation of MMP-9, leading to the release of the CD44 intracellular domain (CD44ICD) [37]. MMP-9 is highly expressed in osteosarcoma [38, 39], in our study, MMP-9 expression was down-regulated after CD44 was knocked down, reflecting the promoting effect of CD44 on invasion and metastasis of osteosarcoma cells, which is consistent with the role of CD44 in other tumours [40, 41].

Cathepsin is secreted on the cell surface in the highly acidic tumour microenvironment and is similar to matrix metalloproteinases in its role in the degradation of multiple extracellular matrix (ECM) proteins and basement membranes, thereby promoting tumour cell invasion and metastasis [42], and a number of cathepsins have been found to activate MMP-9 through the proteolysis of its pro-domain [43]. Cathepsin S (CTSS) is one of a family of cathepsin proteases, which could partially alter the expression of MMP-9 in human corneal epithelial cells through protease-activated [44], MMP-9 and CTSS have synergistic effects in triple negative breast cancer (TNBC), and simultaneous deletion of both can inhibit the invasion of MDA-MB-231 human TNBC cell invasion [45]. CTSS has been found to be associated with a variety of biological functions in cancer. For instance, after the cathepsin S was inhibited, TNBC had a reduced ability to grow and metastasize [45], TGF-β reversed PI3K/AKT/mTOR pathway-induced changed in EMT and tight junction proteins, which in turn inhibit aggressive growth and distant metastasis of glioblastoma [46], the secretion of progesterone and estradiol in rabbit ovarian granulosa cells was regulated and cell proliferation was inhibited. We found that CTSS was also highly expressed in osteosarcoma cells.

In polysaccharide storage myopathy (PSSM), Gene expression analysis showed that CTSS and CD44 genes were more than twofold up-regulated [47], and the expression of CTSS and CD44v9 increased after gastric epithelial injury [48]. Our data showed that after CD44 knockdown, the expression of CTSS in OS cells was consistent with the trend of MMP-9 and also showed downregulation. The regulation mechanism of CD44 in diseases has many crossed signalling pathways with CTSS, such as PI3K/AKT Pathway [49, 50], P38 [51, 52] and ERK1/2-MAPK signalling pathways [53, 54], and other factors like MMP-9 and CD74 [55, 56] are also involved. However, the exact regulatory relationship between the two has not been found in experimental studies. This limitation still needs a lot of work to be further clarified, which may have far-reaching significance for the diagnosis and treatment of osteosarcoma, and we will continue to explore it in the follow-up work.

## Conclusion

This study shows that inhibition of CD44 attenuates cell proliferation, migration and invasion, possibly by regulating the expression of cathepsin S in OS cells. These findings suggest that CD44 may be an oncogenic factor in the progression of OS and may be a promising molecular marker for the diagnosis and treatment of OS.

## Data Availability

The datasets supporting the conclusions of this article are included within the article.

## Abbreviations

OS: Osteosarcoma
CD44: Cluster of differentiation 44
Cathepsin S: CTSS
CCK8: Cell counting kit‑8
MMP9: Matrix metalloproteinase 9
WB: Western blot
RT-qPCR: Reverse transcription-polymerase chain reaction
siRNA: Small interference RNA
